# Perspectives of Executive Nurse Leaders on Advanced Practice Nursing in Saudi Arabia: Challenges to be Overcome and Opportunities to be Seized

**DOI:** 10.1155/2023/6620806

**Published:** 2023-12-11

**Authors:** Arwa Alhamed, Monir M. Almotairy, Ahmed Nahari, Hamza Moafa, Ahmad Aboshaiqah, Fahad Alblowi

**Affiliations:** ^1^College of Nursing, King Saud bin Abdulaziz University for Health Sciences, Riyadh, Saudi Arabia; ^2^Department of Nursing Administration and Education, King Saud University College of Nursing, Riyadh, P.O. Box 642, Saudi Arabia; ^3^Department of Medical Surgical Nursing, King Saud University College of Nursing, Riyadh, P.O. Box 642, Saudi Arabia; ^4^Department of Community and Psychiatric Mental Health Nursing, King Saud University College of Nursing, Riyadh, P.O. Box 642, Saudi Arabia; ^5^Ministry of Health, Riyadh 12382, Saudi Arabia

## Abstract

**Aim:**

To explore the perception of executive nurse leaders regarding the implementation of advanced practice nursing in Saudi Arabia.

**Design:**

An exploratory, descriptive, and qualitative design was used.

**Methods:**

Purposive sampling was used to recruit chief nurse officers and executive nurse directors from government, private, and military health systems in tertiary, secondary, and primary settings. Data were collected from July to November 2022 using virtual semistructured interviews, which were recorded and transcribed. Thematic analysis was conducted manually.

**Results:**

Eleven participants were included in the study. The following themes were identified: unique characteristics of advanced practice nursing, impacts of advanced practice nursing in nursing profession, challenges to utilize the advanced practice nursing roles, opportunities to foster the proper implementation of advanced practice nursing, and recommendations to move forward with fitting advanced practice nursing in Saudi health system. Participants characterized advanced practice nursing as specialized, advanced, and autonomous and reported that it increases access to care, provides efficient and cost-effective care, and expands nursing career pathways. The lack of job description, fragmentation of implementation efforts, lack of training programs, and resistance from physicians, nurses, and patients hinder its implementation. The Saudi Vision 2030, gaps in health care, the growing population, and the familiarity of the healthcare system with advanced practice nursing were viewed as opportunities. The following subthemes emerged under recommendations: establish, train, and implement.

**Conclusion:**

Executive nurse leaders perceived advanced practice nursing as a promising initiative to improve healthcare delivery and the nursing profession. Several opportunities make “now” the right time to implement it.

## 1. Introduction

Healthcare systems continue to transform and expand access to healthcare and improve quality of healthcare services and population health outcomes worldwide [1]. In many countries, the role of nonphysician providers, such as advanced practice nursing (APN), was introduced to reach the objectives for universal health coverage [1, 2] and improve patients' health outcomes [3–6]. However, the implementation of APN in many countries faced challenges and, thus, necessitated actions to facilitate proper implementation [7–9]. An international study sought to examine the implementation status of advanced practice nursing regulations globally, and they found wide variation in educational requirements, regulations, and scope of practice in 26 countries from North America, Europe, Asia, South America, and Africa [9]. Out of 16 countries that implemented nurse practitioner (NP) role, 9 countries had the NP role regulated. Out of 13 countries that implemented clinical nurse specialist (CNS) role, only 6 countries had the CNS role regulated [9]. Out of 10 countries that implemented general APN roles without specifying whether it is NP or CNS, only 5 countries had the APN regulated [9].

The APN is evolving in the Middle East countries [8], including Saudi Arabia [10], and there is no clear evaluation of potential impacts of the APN roles on organizational and patients' health outcomes. According to Almukhaini et al. [8], who summarized evidence regarding advanced practice nursing roles in Arab countries located in the Eastern Mediterranean region, the main drives to implement the APN were the shortage of physicians, increasing incidences of chronic diseases, to expand cost-effective access to primary care, and promote the nursing profession. The main barriers to implement the APN in the Middle East were “a lack of recognition of roles at national levels, role ambiguity, lack of clear scope of practice, resistance from male physicians, low involvement of nurses in policy-making, and low status of nursing as a profession” [8].

In Saudi Arabia, little is known about facilitators and barriers to implement APN. Previous studies tried to unveil different aspects relevant to facilitating the implementation of APN in the Saudi healthcare system [10–12]. In contrast to some findings from Almukhaini et al. [8], a recent study found that physicians in Saudi Arabia support the implementation of nurse practitioner roles in primary care settings [12]. For instance, another study identified that APN tasks and activities are existing in Saudi Arabia, which warrant regulatory delineation actions to facilitate the implementation of APN roles [10].

Executive nurse leaders are nurses in “executive practice who set the vision for nursing practice in the delivery of safe, timely, efficient, equitable, and patient-centered care” (p.2) [13]. Executive nurse leaders play a significant role in facilitating the implementation of APN in healthcare systems by identifying and advocating for organizational and national health policy reforms [14, 15]. Various studies that exist on the perspectives of nurse leaders regarding the implantation of advanced practice nursing roles internationally have shown that nurse leaders can better evaluate the potential of fitting APN roles within their healthcare system and overcome potential barriers and challenges [16, 17]. To the best of our knowledge, the perspectives of executive nurse leaders regarding the feasibility of implementing APN have not been investigated in Saudi Arabia. Insights from executive nurse leaders regarding the current situation and progress toward implementing APN in Saudi Arabia can guide efforts to facilitate effective implementation and reduce potential barriers at organizational and practice levels. The perspective of executive nurse leaders in Saudi Arabia can contribute to the international dialogue on best practices for the APN roles' implementation. Thus, this study explored the perception of executive nurse leaders regarding APN definitions, impact, challenges, opportunities, and facilitators for implementing APN in Saudi Arabia.

## 2. Materials and Methods

### 2.1. Design

This study utilized an exploratory, descriptive, and qualitative design. The consolidated criteria for reporting qualitative research (COREQ) were followed to report the findings of this study.

### 2.2. Setting and Recruitment

This study was conducted from July to November 2022 and recruited executive nurse leaders from government, private, and military health systems in tertiary, secondary, and primary healthcare settings in Saudi Arabia.

### 2.3. Inclusion and/or Exclusion Criteria

This study recruited nurses serving as top-level nurse leaders, such as chief nurse officers and executive nurse directors, who are usually involved in shaping future directions of nursing in their health organizations. Thus, middle-level nurse administrators, such as nurse supervisors and head nurses, were excluded from the study.

### 2.4. Data Collection

Virtual individual semistructured interviews were conducted (Supplemental Table 1). First, email invitations, including an introduction to the study, were sent to eligible participants. The email invitation asked participants to suggest convenient time slots for the conduction of interviews. Those who agreed to participate in the study received another email confirming the interview time and a secure link for a virtual meeting (Zoom or Microsoft Teams). At the beginning of each interview, participants were informed that the interview would be recorded for data analysis purposes and that they needed to speak in English for more accurate transcription. In addition, participants were identified using a study code which they used to complete the demographic survey. The participants were provided with a survey link for demographic data, which included gender, highest level of education, country of education, overall years of experience, years of experience in leadership, presence of APN degree and executive leadership qualifications, type of work institution, and whether their institution implemented APN. This was followed by interviews using seven open-ended questions. Data saturation was reached with seven participants; however, additional participants were recruited so as to include different systems and cities, which resulted in 11 participants.

### 2.5. Data Analysis

Descriptive statistics were used to describe the demographic characteristics using SPSS version 28 (SPSS Inc., Chicago, IL, USA). Thematic data analysis followed Braun and Clarke's approach [18]. All recordings were deidentified, saved on secured cloud storage, and transcribed verbatim by a professional transcription agency. Two authors, including the first author, checked the transcripts against the recordings for accuracy. Two authors read and reread the transcriptions individually to draw codes. One author used NVivo (QSR International, 2020), and the other conducted manual coding. The two authors grouped the generic and main codes into broader themes. Subsequently, the two authors met to discuss the findings and agreed on 95% of the codes and themes. The remaining 5% were integrated into other broader themes. The interpretation of findings and relevant quotes were discussed with other authors not involved in data collection, transcription, and analysis.

### 2.6. Ethical Considerations

This study was approved by the appropriate Institutional Review Board at King Saud University (IRB Reference number: 22/0594/IRB, IRB approval date: April 25^th^, 2022, Research project no. E-22-6845). Individuals who indicated their interest to participate in the study received an email including the informed consent and the demographic data survey links. The informed consent included an introduction to the study, a description of the data collection methods, the description of the risks and benefits of participation in the study, and the participants' option to withdraw from the study at any time. Participants were requested to open the link and indicate their agreement to participate in the study before they started to complete the demographic survey and before the beginning of the interview.

### 2.7. Rigor and Reflexivity

The study followed several structured steps to maintain the credibility and confirmability of the analysis and findings. First, most authors were experts and specialized as APNs, whereas others were experts from regulatory and clinical fields. Second, the authors' expertise facilitated the development and refinement of the interview questions to address the regulatory and operational aspects of implementing APN. Third, initial findings were discussed to reach a consensus on the codes and themes which emerged from data collection. Fourth, study findings were reported according to the COREQ guidelines [19]. Lastly, the authors provided rich, detailed descriptions of all aspects of the phenomenon under investigation to address transferability.

## 3. Results

The sample's demographic characteristics are presented in Table 1.

The following themes were identified: unique characteristics of advanced practice nursing, impacts of advanced practice nursing in nursing profession, challenges to utilize the advanced practice nursing roles, opportunities to foster the proper implementation of advanced practice nursing (Table 2), and recommendations to move forward with fitting advanced practice nursing in the Saudi health system (Table 3).

### 3.1. Unique Characteristics of Advanced Practice Nursing

Most participants only identified the NP role when describing the APN, and they used the two terms interchangeably. However, few participants could list additional APN roles, which were mainly nurse specialist, nurse anesthetist, and nurse practitioner (NP). Participants described APN as more specialized, requiring advanced education, training, and skills such as decision-making, diagnosing, interventions, and collaboration, and with the ability to practice with more autonomy compared to registered nurses. One participant described APN as follows:

*“Who deliver a care to a patient with specialized care, requiring high technical skills, someone who is more specialized in his specialty from either program or experience.” #116*

Moreover, participants referred to APNs as a link between the physician and the registered nurse. One participant described this as follows:

*“The one who is between a registered nurse and the physician.” #0114*

Participants viewed APN roles as distinct as they fill specific gaps in care rather than replace physicians, as one participant stated

*“Not because we have a higher number of NPs, we will not hire MDs, nurse practitioners have different roles than physicians.” #0111*

### 3.2. Impacts of Advanced Practice Nursing in Nursing Profession

Participants reported several positive impacts of APN on patients, organizations, and nursing if implemented in Saudi Arabia. Participants reported that implementing APN can increase access to care and reduce waiting time, as one participant stated

*“As you can see, we have many patients coming, sometimes waiting for hours to see the doctors. But if we have a nurse practitioner, at least they would see those patients much faster.”* #0115

Participants described that APN roles are stemming from nursing background, so a holistic care approach across lifespan and a range of conditions was described, by participants, as an impact of APNs. One participant stated that

*“NPs can provide holistic nursing care to any population.” #0118*

Participants identified several positive impacts on organizational outcomes. For instance, participants indicated that implementing the APN can enhance as patient satisfaction, experience, and safety, as one participant explained

*“So, they would be very close to the patient because it would improve their satisfaction and patient experience.” #0110*

In addition, executive nurse leaders, in line with the literature, thought that the APN roles are efficient and cost-effective for hospitals, yet not a midlevel and undervalued service, as participants stated

*“Yes, you can have a great impact in the organization because of the cost effectiveness.” #0119*

*“We are not a second-class service.” #0117*

Moreover, participants shared the perspective on a potential impact that APNs can relieve physicians from the load of seeing low-acuity patients to focus on critical cases, as a participant stated

*“We would complement physicians” role so they can focus on more serious cases.” #0117*

Additionally, participants revealed that APNs could fill several gaps in healthcare related to the shortage of physicians, as a participant stated

*“In some parts of the world where there is a significant shortage of physicians, the only way to fill the gap was looking for APNs.” #0115*

Participants suggested that the establishment of APN will expand nursing career pathways and attract more students to the nursing profession. A participant explained

*“The number one desire for nurses is the ability to grow, and to professionally develop… this would definitely provide an opportunity that has never been available to them before.” #0115*

### 3.3. Challenges to Utilize the Advanced Practice Nursing Roles

Participants had mixed viewpoints regarding the readiness of the current healthcare system to implement APN. Few participants viewed the system as “*not ready now, but has the capability to*” (#0110), whereas others viewed the system as *“ready” (#0112).* However, all participants agreed on common regulatory, professional, academic, and cultural challenges hindering APN implementation. Furthermore, participants reported registration and credentialing-related challenges. They discussed that regulatory bodies do not recognize the APN title in Saudi Arabia and do not have core competencies for credentialing or scopes of practice, as one participant stated

*“We do not have a standardized job description for APN, each institution creates their own, but it is not approved by higher management. So, it is not clear, not certain.” #0111*

As such, participants indicated that current APN implementation was institution-based, informal, inconsistent, and provided unfair pay and no malpractice insurance. A participant explained

*“If the role privileges are based on an internal agreement within the organization, they can be impacted by any change. But if it is based on the regulatory body, such privileges will be consistent.” #113*

Moreover, most participants indicated that the current nursing professional classification framework is generic (classifies nurses based on their level of education rather than their specialty), which limits the expansion of nursing specialties, a core characteristic of APN. One participant explained

*“A nurse with a diploma or a bachelor's degree will be classified as a specialist, a nurse with a master's degree will be classified as a senior specialist, and those with a doctorate degree will be classified as consultants, there is nothing else.” #0114*

Participants highlighted the efforts of several clinical and academic entities to facilitate the implementation of APN. However, such efforts were viewed as fragmented, inconsistent, down-top, and lacking the involvement of all stakeholders, as one participant stated

*“I think we have fragmentation in our regulatory body. We have full body, with arms and legs and so on, but everything is working on his own way.” #0111*

Several participants indicated that there was a need for more local APN academic programs. A few local academic institutions have developed APN programs; however, due to the lack of senior APN nurses and physicians available to train and the lack of standard credentialing requirements, the competency of the graduates was questioned. A participant stated

*“They have the theoretical, but they did not have the practice knowledge.” #0116*

Resistance from physicians and nurses offered APNs a challenge, as one participant explained

*“You are perceived as a threat to physicians and some nurses felt that we are above them.” #0117*

Some participants indicated that APN might face resistance from patients since APNs will assume roles that are conventionally known to be physicians' roles. One participant explained this as follows:

*“I think the level of acceptance by patient will probably be one of the challenges because until today, all aspects of care are under the physician.” #0118.*

### 3.4. Opportunities to Foster the Proper Implementation of Advanced Practice Nursing

Participants identified several opportunities that may catalyse the implementation of APN. Saudi Vision 2030 was viewed as an opportunity to implement APN, as one participant explained

*“We can have the changes and the 2030 vision will have a positive impact on this.” #0113.*

Participants viewed the gap in primary care as an opportunity, and APNs will fill the gaps, as one participant explained

*“The biggest piece of the model is going to be focused on primary care…, all physicians go for sub-specialties… So, this is where the role of APN comes….” #0115*

From an organization perspective, participants viewed the care provided by APNs as efficient and cost-effective, as one participant explained

*“Once you have highly qualified APN, nurses will be recognized as revenue generators.” #0115*

Physicians were perceived to be less resistant to APN due to their North American training, as one participant explained

*“I have here a number of our medical team were trained in Canada and the States, and they know about the role and everything, and the majority of them are supporting.” #0112*

In addition, participants viewed North American hospital accreditation standards as enforcers of APN, as one participant explained

“*We do not have a nurse practitioner, but we are midway in our journey to get to Magnet recognition. So, one of the initiatives within the organization is to have a clear regulation on nursing practitioner.” #112*

Furthermore, participants viewed the COVID-19 pandemic as an opportunity to expedite the implementation of APN, as one participant stated

*“I think if we had nurse practitioners during the COVID-19 pandemic, they would have been a great relief to the system, and it would have been the best facilitator to implement the APN role.” 0113*

### 3.5. Recommendations to Move Forward with Fitting Advanced Practice Nursing in Saudi Health System

Participants agreed on several recommendations to facilitate the implementation of APN in Saudi Arabia. These emerged under three main subthemes: establish, train, and implement (Table 3).

#### 3.5.1. Establishment of APN Roles

Participants agreed that establishing APN at the regulatory level is the first step toward implementing APN. Participants indicated that the efforts of all stakeholders, such as nursing clinicians, academicians, and policymakers (i.e., Saudi Commission for Health Specialties, Ministry of Health, Ministry of Education, Ministry of Human Resources, Scientific and Professional Councils) need to be integrated to establish APN roles. A participant explained this as

*“I think there has to be some type of an advisory committee, with all different parts of legislative and education, come together as a group, not fragmented.” #0115.*

In addition, participants reported that data from current APN implementation models could help guide the effective implementation of APN. A participant stated

*“There is a lot of evidence showing the positive impact of having a nurse practitioner, we can present this evidence to leaders.” #0113*

Participants highlighted the need to introduce APN to the community as one participant indicated

*“I need to educate the community, also the healthcare providers and the organizations from leadership starting from our regulatory bodies (i.e., The Saudi Commission for Health Specialties).” #0113*

Stakeholders need to develop clear and standard APN job descriptions, scopes of practice, core competencies, and credentialing requirements. One participant noted

*“We need to define scope with competencies, certain privileges, certain protocols.” #0110*

#### 3.5.2. Training Required for Proper Implementation

Participants recommended the continuation of international scholarships to train APNs:

*“We can't stop scholarships; we need to go on that.” #0110*

In addition, participants suggested establishing academic partnerships with international academic institutions to offer APN training programs:

*“We need to look into blended learning or collaborative learning, where students can be taught by international institutions in national universities through academic partnerships.” #0110*

Moreover, participants suggested developing local APN education programs utilizing the qualifications and experience of existing APNs to train:

*“There are nursing colleges in the kingdom and are working to establish APN programs. Their professors were already trained in North American facilities, and they were exposed to having such program within the academic and with collaboration with hospitals.” #0112.*

Participants agreed that admission requirements for APN programs need to be standardized such as a minimum of a Bachelor of Nursing degree, as one participant stated

*“Someone who has graduated with a bachelor of nursing degree and is registered as registered nurses.” #0112*

Furthermore, there should be a minimum number of years of experience, as one participant stated

*“It is pretty important that they have the clinical experience before they are admitted to APN degree.” #0112*

Most participants indicated that 2 to 3 years of experience in a specific nursing specialty should be the minimum requirement to be accepted into an APN degree as one participant stated

*“Two years of experience in a specific role, I think, is enough for a person to be given a key to go for a specialized role as an APN.” #0116*

However, some participants expressed concerns regarding the limited ability to ensure that years of experience alone reflect the experience required for APN, alluding to the discrepancy in nursing roles across institutions and settings across the country. One participant stated

*“But how can we validate the experience, we have discrepancies here which could be different from one hospital to another.” #0112*

Most participants recommended that academic programs be clinically based in parallel to theoretical education, as one participant stated

*“I would not recommend somebody go off and sit in a classroom and to practice on a mannequin. No, they need to be doing it within the clinical area.” #0117*

*“It must include 60 to 70% clinical training.” #0111*

Moreover, participants recommended the establishment of partnerships among clinical and academic settings to build the capacity to train and hire APNs.

#### 3.5.3. Implementation Approach of APN

Participants indicated the need to establish a clear organizational structure incorporating APN. While all participants indicated that APN should be under nursing administration, the reporting, credentialing, and evaluation of APNs in an organization should be a joint task between nursing and medicine, as one participant stated

*“I believe they should report to advance practice nursing department which should report to the chief nursing officer, with involvement of the medical side.” #0111*

Participants stressed the need to set clear and standardized payments, reimbursements, and liability insurance guidelines. Additionally, participants highly stressed the need to establish a collaborative agreement with physicians and seek their support to facilitate the implementation of APN as one participant stated

“*You will need to collaborate and establish partnerships with physicians to be able to do your role.” #0114*

Furthermore, participants indicated that utilizing the qualifications and experience of existing APNs in role development is essential, as one participant stated

*“We have those groups of nurse practitioner already graduated; they are available to assume the role immediately.” 0111.*

Participants suggested that APN can be initially implemented in primary care settings:

*“We can start on a specialty basis. So, we can start with primary care.” #0115*

## 4. Discussion

This study explored the perception of executive nurse leaders regarding APN, the major finding themes were the following: unique characteristics of advanced practice nursing, impacts of advanced practice nursing in nursing profession, challenges to utilize the advanced practice nursing roles, opportunities to foster the proper implementation of advanced practice nursing, and recommendations to move forward with fitting advanced practice nursing in the Saudi health system. Participants in our study provided inconsistent identifications of APN roles, and the majority referred to APN as nurse practitioners (NPs). In addition, participants provided inconsistent entry-level training requirements for APN. This finding was consistent with previous reports indicating longstanding inconsistencies in the definitions of APN, their scope of practice, and entry-level training requirements worldwide [9, 11, 15, 20]. Such inconsistency was viewed by the International Council of Nurses (ICN) as one of the most significant challenges hindering the implementation of APN and called for the need to standardize definitions, scope of practice, and clear entry-level education for APN [21]. Nevertheless, participants agreed on the main characteristics of APN such as advanced training and skills, specialized knowledge and experience, and autonomous practice. Such characterizations agreed with the APN definitions of the Canadian and the American Nurses Association [22, 23].

Consistent with previous reports [24, 25], this study indicated that some forms of APN were implemented in Saudi Arabia. The current implementation model involves the development of institution-specific role descriptions and credentialing requirements. Such an implementation model needs to be more structured, consistent, and recognized by regulatory bodies. While it has been viewed as a promising effort, such unclear delineation of APN raises several health, professional, organizational, financial, and legal concerns [10, 24].

Nevertheless, participants identified several patient-related, organizational, and professional positive impacts of APN in Saudi Arabia. Similar findings were reported in recent studies in Saudi Arabia [25–27] and worldwide [28–31].

Several challenges facing the implementation of APN in Saudi Arabia were identified in this study. Similar to previous studies, participants indicated that the lack of legislation hinders efforts to implement APN in Saudi Arabia [9, 10, 15]. Moreover, participants highlighted the need for well-developed local academic APN programs with adequate clinical training for safe and independent practice. While the American Association of Nurse Practitioners [32] indicated that a master's degree is required at the entry level to APN, consensus on educational programs was not easily achieved [33]. In addition, participants indicated that APN may face resistance from physicians, nurses, and patients, which was consistent with previous studies [34]. However, recent studies revealed that physicians [12] and nurses [26, 27] in Saudi Arabia supported the implementation of APN. To the best of our knowledge, no studies have explored the patients' experiences with the care provided by APNs. Future studies on this topic are therefore recommended.

Participants in this study identified several opportunities that could catalyse the implementation of APN in Saudi Arabia. The Healthcare Transformation Program (HTP), one of the Saudi Vision 2030 programs, aims to restructure the healthcare system to improve healthcare delivery and the population's health [35]. Several objectives have been set to reach such goals, such as improving the quality and quantity of the Saudi nursing workforce. As such, participants believed that implementing APN would expand career pathways and increase the attractiveness of the nursing profession [33]. In addition, the HTP aims to enhance access to primary healthcare. Moreover, the prospective increase in the geriatric population in Saudi Arabia will create additional demands on the existing healthcare system. Therefore, existing gaps in care will be magnified, and APNs may fill such gaps in care [31].

Furthermore, the HTP aims to expand the privatization of healthcare. Participants viewed the care provided by APNs as efficient and cost-effective, making employers eager to hire them [30]. As such, APNs can be utilized to help achieve the objectives of the HTP.

Many physicians in Saudi Arabia were trained in the USA and Canada and are familiar with APN. Hence, participants viewed physicians as less resistant to implementing APN, consistent with previous reports from Saudi Arabia [12]. In addition, hospitals in Saudi Arabia have been recently moving toward obtaining institutional accreditation by North American organizations such as Magnet [36], and such movements may lead to the development of national regulation for APN by regulatory bodies.

Pandemics can reveal potential opportunities to expand the access to care. For instance, participants in this study viewed the COVID-19 pandemic as an opportunity to expedite the implementation of APN. Many institutions reviewed and restructured their care delivery systems to meet the shortage of healthcare providers triggered by the pandemic. APN can be implemented to meet similar needs. A similar situation was reported in the USA [37], accelerating APNs' licensing and expanding their scope of practice [38].

The implementation of APN in Saudi Arabia warrants crucial planning as recommended by participants. They indicated that the first step toward implementing APN was establishing their role through regulatory bodies using a top-down approach. A similar approach was utilized in other countries where APN was not well developed [39], and the ICN has recently called for such an approach [21]. Participants viewed existing efforts by regulatory, clinical, and academic entities as fragmented and counterproductive. They recommended integrating efforts from all stakeholders (i.e., Ministries of Health, Ministry of Education, Ministry of Human Resources and Social Development, and the Saudi Commission for Health Specialties) through an APN advisory board. The development of clear and standard APN role descriptions, scopes of practice, core competencies, and credentialing requirements is necessary toward fitting the APN within the Saudi healthcare system. When policies defining APN roles and scopes of practice need clarity, healthcare administrators will be skeptical and resistant to implementing the role [31]. Clear policies guide role implementation and education establishment [40].

The entry requirements for APN need to be standardized to ensure the quality of potential practitioners in the APN roles. For instance, most participants agreed that admission requirements for APN programs must be standardized, such as a minimum of a Bachelor of Nursing degree and 2 to 3 years of experience in bedside nursing [32]. In addition, they stressed the need to ensure that academic programs include intense clinical training in parallel to theoretical education. However, participants indicated that measuring clinical experience using hours and/or years of clinical training is not enough to reflect the quality of training required for APN. Previous reports indicated that the current APN educational model using credit hours of clinical training is no longer effective. Instead, competency-based training is recommended with the utilization of simulation when specific learning experiences are not available in clinical settings. Such an approach ensures standardization and consistency in training and evaluation [41, 42].

The establishment of partnerships between clinical and academic settings to build the capacity to train and hire APNs was also recommended by participants. Partnerships between nursing and healthcare institutions ensure clinical placement, facilitate faculty practice, and connect students with potential employers [43]. Moreover, administrative policies and procedures regarding whom APNs report to, who credentials them, and who evaluates their performance need to be established. While all participants indicated that the APN roles should be under nursing administration, the reporting, credentialing, and evaluating of APNs in an organization should be a joint task between nursing and medicine. Implementing distributed leadership is a practical management approach for interconnected jobs such as APN and medicine [44]. In addition, such an approach effectively sustains collaborative relationships between nursing and medicine.

As part of the implementation of APN roles, there is a need to set clear and standardized guidelines for payment, reimbursement, and liability insurance for APN, as the lack of such guidelines hinders the implementation of APN [45]. Moreover, as there is a shortage of physicians in primary care, physicians in Saudi Arabia supported the implementation of APN in primary care [12]; hence, participants suggested that the APN role can be initially implemented in primary care. Increasing emphasis on primary and community health care stimulated the appearance of the APN role in the USA and Canada [33].

Community awareness about the potential benefits of APN on health outcomes can play a significant role in fostering the implementation of APN roles. Thus, it is important to introduce APN to the community to prepare them for the expected paradigm shift in care delivery if APN was to be implemented [46]. Nursing societies can conduct national awareness campaigns with the Ministry of Health and the Saudi Commission for Health Specialties. Such awareness campaigns should be guided by available international evidence related to the positive impact of APN [34, 47].

Overall, participants highlighted the role of three interconnected factors behind change in large organizations such as healthcare suggested by Pettigrew et al. [48], the presence of environmental pressure (e.g., Vision 2030, COVID-19), a supportive organizational culture (e.g., North American Trained Physicians and hospital accreditation standards), and effective managerial-clinical relationships (e.g., collaboration and partnership) [49].

To the best of our knowledge, this is the first study to examine the perceptions of executive nurse leaders in the Arab region regarding the feasibility of implementing APN. Our study included leaders from diverse healthcare systems in Saudi Arabia, including public, private, and military systems in primary, secondary, and tertiary healthcare settings. This diversity increased the credibility of our findings. However, this study has some limitations. Only some hospitals partially implemented APN in Saudi Arabia which may have affected our ability to capture all the possible challenges facing the implementation of APN. Future research should consider including nurses from different levels of leadership to obtain a clearer understanding of the perception and readiness of the hospitals in Saudi Arabia to implement APN. In addition, exploring the patient's experience with the care provided by APN may enhance the understanding of the benefits and challenges of implementing APN in Saudi Arabia. Further research is needed to better understand the readiness of primary healthcare centers to implement APN.

## 5. Conclusions

Executive nurse leaders perceived APN as a promising initiative to improve healthcare delivery, promote professional development, and improve patient care. Opportunities such as the Healthcare Transformation Program under the Saudi Vision 2030, gaps in healthcare magnified by the COVID-19 pandemic and the growing population, and the familiarity of the Saudi healthcare system with APN roles due to the North American training of the physicians and the adoption of North American hospital accreditation standards make it an ideal time to implement APN. Therefore, integrating efforts from all stakeholders through a national board for APN is essential to develop and standardize regulations for APN in Saudi Arabia.

## Figures and Tables

**Table 1:** Demographic characteristics (*n* = 11).

Variables	Category	Frequency (%)
Gender	Male	7 (63.6)
	Female	4 (36.3)

Highest level of education	BSN	1 (9)
	Diploma	1 (9)
	Master	7 (63.3)
	PhD	2 (18)

MSN country	USA	3 (42.8)
	UK	1 (14)
	Australia	1 (14)
	Saudi Arabia	2 (28)

PhD country	UK	2 (100)

Overall years of experience	10–20	2 (18)
	20–30	6 (54)
	>30	3 (27)

Years of experience in leadership	≤5	1 (9)
	6–10	3 (27)
	11–20	3 (27)
	≥20–30	4 (36)

APN degree	Yes	3 (27)
	No	8 (72)

Type of APN specialty	Pediatric and adults (dual)	1 (33)
	Geriatric	2 (66)

Executive degree in leadership	Yes	6 (54)
	No	5 (45)

Country of education	USA	3 (50)
	UK	1 (16)
	Others	2 (33)

Work institution type	Private	1 (9)
	Tertiary	7 (63)
	Health cluster	3 (27)

APN role implemented	Yes	5 (45%)
	No	6 (54)

*Note.* APN: advanced practice nursing; BSN: Bachelor of Science in Nursing; MSN: Master of Science in Nursing.

**Table 2:** Characteristics, impacts, challenges, and opportunities for implementing APN (*n* = 11).

Themes	Subthemes	Codes	Subcodes
Unique characteristics of advanced practice nursing	Specialized	Depth in knowledge and experience in a specific area	Cardiac, oncology, midwifery,
	Advanced	Education	A postgraduate diploma
			A master's degree, doctorate in nursing practice
		Training	Clinical training
		Skills	Diagnoses, health assessment, ordering diagnostic tests, and prescribe medications
	Autonomous	Run their own clinics, practice independently, privileges	
	A link between physicians and nurses		
	Distinct role	Not replacing physicians	

Impacts of advanced practice nursing in nursing profession	Access to care and reduced waiting time	Emergency room, outpatient	
	Patient satisfaction and experience		
	Patient safety		
	Holistic care and efficiency	Patient-provider relationship	
		Adequate health education	
		Follow-up	
	Relief physicians	Focus on critical cases and subspecialties	
	Cost-effective	APN are paid less than physicians APNs will be revenue makers	
		Do not provide low-quality care	
		Do not abuse the role	
	Fill gaps in care	Primary, rural, school health, chronic disease management	
	Expand nursing career pathways	Retain and attract nurses	
		Concerns regarding worsening shortage of registered nurses	

Challenges to utilize the advanced practice nursing roles	Regulatory		
	Professional	Resistance from physicians and nurses	
	Academic	Lack of programs	
		Existing programs lack clinical training	
	Cultural	Resistance from patients	

Opportunities to foster the proper implementation of advanced practice nursing	Saudi Vision 2030	Enhance the quality and quantity nursing profession	
		Existing gaps in care	
		Privatization	
	North American models	Training	
		Hospital accreditation	
	COVID-19 pandemic		

**Table 3:** Recommendations for implementing APN.

Subthemes	Codes	Subcodes
Establishment of APN roles	Who?	Involve all stakeholders
	How?	Integrate efforts
		Utilize existing APN
		Utilize data from existing models
		Introduce the APN role to the community
	What?	Role description
		Scope of practice
		Competencies and credentialing

Training required for proper implementation	Academic programs (international, hybrid, or local)	Clinical/specialty based
	Standardize admission requirements	Minimum years of experience
	Establish partnerships	Universities and hospitals
		Nursing and medicine

Implementation approach of APN	Establish APN organizational structure	Reporting, training, evaluating, and credentialing
	Set guidelines for financial compensation	Pay, reimbursement, and malpractice insurance
	Establish partnerships	To establish clinical training and jobs
	Utilize existing APN	To develop programs and train
	Start with most needed specialties	Primary care

*Note.* APN: advanced practice nursing.

## Data Availability

The data that support the findings of this study are available on request from the corresponding author and are not publicly available due to privacy or ethical restrictions.

## Additional Points

*Implications for the Profession and Patient Care*. Implementation requires establishing advanced practice nursing at a regulatory level, developing a standard job description and training programs, incorporating it in organizational structures, and implementing advanced practice nursing in primary care. *What does this paper contribute to the wider global clinical community?* This study may guide advanced practice nursing implementation efforts in countries where advanced practice nursing is not well developed.

## Supplementary Materials

Supplementary Materials Supplementary Table 1 presents the qualitative questions and prompts used during the interview. The questions and prompts are presented sequentially according to the role implementation-related domains.
