# Supplementary material for: Perspectives of Executive Nurse Leaders on Advanced Practice Nursing in Saudi Arabia: Challenges to be Overcome and Opportunities to be Seized
Source: J Nurs Manag. 2023 Dec 11;2023:6620806. doi: 10.1155/2023/6620806 (PMC11918968; doi:10.1155/2023/6620806)
Supplement: Supplementary Materials — Supplementary Table 1 presents the qualitative questions and prompts used during the interview. The questions and prompts are presented sequentially according to the role implementation-related domains. [file 6620806.f1.docx]

**Supplementary file 1**

**Interview Schedule**

| Items | Interview questions |
| --- | --- |
| Before starting the interview | Could you please introduce yourself |
|  | Before interview, we would like to ask about some demographic information. If that is fine, please check the chat and click on the link to complete several questions. You would be asked about your assigned code, which we would use to de-identify collected data. Thus, please use this code (XXX). Once you are done with the survey, please let me know, so we can start the interview. |
| General Perspective | - How do you define or describe APN? - Probing question: (if participant focus on one APN role, then ask this question):   - What do you know about other APN roles? |
| Questions about the practice | What do you think are the differences between the APN roles and the RN roles?  How do you think the APN/NP role (depends on how participant defines APN) would impact the delivery of care in Saudi Arabia?  Probing question:  What about its effect on patient outcomes, organizational outcomes?  What do you think are the main challenges facing the APN roles in the clinical settings in Saudi Arabia and in your institution?  Probing questions:  What about the scope of practice?  What about the practice authority and privileges?  How do you see the relationship of APNs with physicians in term of the practice? (e.g., collaborative agreement)?  What about the classification form Saudi Commission for Health Specialties?  What do you think are the main facilitators for introducing the APN roles in the clinical settings in Saudi Arabia? |
| Questions about education, regulation, legislation requirements | - How do you see the future of the APN roles in Saudi Arabia? - Probing question:   - How do you think we can ensure the adequate supply of APNs in Saudi Arabia? (e.g., scholarships?) - What do you think should be the educational and training requirements to be an APN roles? - Probing questions:   - What do you think the requirements to be admitted into APN academic programs? (Minimum years of experience, specialty)? What about in your institution? Are those requirements implemented?   - What are your thoughts about post-academic programs training for APNs? (e.g., residency and fellowship programs and scope)?   - What do we need at the legislation level to promote the APN roles in the Saudi healthcare system? What do you think about the regulation requirements for APN classifications, certifications, and licensure as a nurse practitioner? What about the role of the Saudi Health Council? the Scientific council, the Professional council? - Let say that your hospital/institution has/want to implement an APN role (e.g., can use NP as an example), how do you envision the APN/NP role within the organizational structure? - *Probing questions:*    - What changes do you expect to happen within the nursing administration department? Do you think the APNs will be under the nursing administration department or other departments? |
| The end | Do you have any additional thoughts that you would like to say toward any of our previous questions? Overall suggestions to successfully implement advanced practice nursing in Saudi Arabia? Or anything you would like to add?  Thank you for dedicating time out of your busy schedule to participate and share your insight about advanced practice nursing. |
